# Engaging with Culturally and Linguistically Diverse Communities to Promote Palliative Care That Exceeds Expectation

**DOI:** 10.1111/hex.70089

**Published:** 2024-11-04

**Authors:** Ann Dadich, Gregory Crawford, Peter Laintoll, Issac Zangre, Kamal Dahal, Dalia Albrezi, Cathie Jeffs, Aileen Collier

**Affiliations:** ^1^ School of Business Western Sydney University Parramatta New South Wales Australia; ^2^ Faculty of Health and Medical Sciences Northern Adelaide Local Health Network University of Adelaide Adelaide South Australia Australia; ^3^ Community Member Adelaide South Australia Australia; ^4^ Northern Adelaide Palliative Service Adelaide South Australia Australia; ^5^ College of Nursing and Health Sciences Flinders University Adelaide South Australia Australia

**Keywords:** brilliant care, culturally and linguistically diverse communities, death, end‐of‐life care, knowledge translation, palliative care, POSH‐VRE

## Abstract

**Introduction:**

Given longstanding barriers that obstruct integrated palliative care, particularly for culturally and linguistically diverse communities, this article demonstrates a way to engage with Syrian, Bhutanese and African communities to learn about brilliant palliative care with and from members of these communities.

**Methods:**

This study involved the methodology of POSH‐VRE, which combines positive organisational scholarship in healthcare (POSH) with video‐reflexive ethnography (VRE). Members of the Syrian, Bhutanese, and African communities (*n* = 14) participated in a focus group or an interview to consider understandings of palliative care; conceptualisations of a good death; how and why palliative care was typically enacted in their communities; the associated effects; as well as the relationship between culturally and linguistically diverse communities and public palliative care services. Discussions were aided by video recordings captured during the previous study on brilliant palliative care, which participants were invited to review. Video recordings and transcripts of the focus groups and interview were analysed using reflexive thematic analysis.

**Results:**

The participants demonstrated considerable variability in the ways that palliative care was understood and enacted. For some, death was a taboo topic, while for others, it was a reality that was required to face, particularly in war‐torn regions. Similarly, while doctors were held in high regard, participants held different views about how they should enact palliative care and the anticipation of death, particularly because family members were deemed to be a pivotal part of palliative care. To improve the care of people of culturally and linguistically diverse communities who experience a life‐limiting illness, participants highlighted three opportunities. These included the avoidance of generalisations, prioritising the needs and preferences of cultural groups, and leveraging the community network.

**Conclusion:**

This study demonstrated how reciprocal understandings of palliative care were potentiated using POSH‐VRE. Specifically, the members of the Bhutanese, African and Syrian communities demonstrated diversity in the needs, preferences, and customs of culturally and linguistically diverse communities. As such, integrated palliative care is likely to be bolstered by relinquishing assumptions about how cultural groups wish to be referred to and cared for and adopting a public health approach to palliative care that embraces both a population‐based and person‐centred approach to care.

**Patient or Public Contribution:**

Members of the Bhutanese, African and Syrian communities contributed to this study as participants and co‐researchers, contributing to the analysis and interpretation of the data and in the preparation of the article.

## 1 Introduction

Palliative care is ‘an approach that improves the quality of life of patients… and their families who are facing problems associated with life‐threatening illness’ [1]. Palliative care is not disease‐ or condition‐specific, nor is it a treatment or an intervention—instead, it is ‘an approach’ that extends across different health issues, irrespective of age or culture.

Palliative care is arguably a context in which the integration of care is most important for patients, families, and communities with palliative care needs. This is largely because care is often provided by informal carers, such as family members, who negotiate with multiple specialists and the associated health and social service providers who are situated across the private, public, and not‐for‐profit sectors [2]. This reflects van der Eerden et al.'s [3] conceptualisation of integrated palliative care as the coalescence of ‘administrative, organisational, clinical and service aspects… to realise continuity of care between all actors involved in the care network of patients receiving palliative care’.

Facilitating integrated care can be complicated and challenging [4]—this extends to palliative care [5]. This is partly because of the disparate understandings and priorities among service providers, policymakers, patients, and family members [6]. For instance, the Royal Commission into Aged Care Quality and Safety highlighted the fragmented care of older Australians [7]. This included the number and complexity of care providers across commonwealth and state jurisdictions as well as siloed health and aged care services.

The challenges of integrated care are exacerbated by the health disparities among people requiring palliative, end‐of‐life, and bereavement care [8, 9, 10]. For instance, an Australian government report highlighted that people of culturally and linguistically diverse backgrounds experience a range of barriers to palliative care [11]. These include the availability of few culturally appropriate resources, the challenge that service providers experience when accommodating cultural practices, patients' and family members' limited trust in services, discrimination, and cultural stereotyping. These inequities are rarely accounted for in integration strategies. For instance, following their analysis of 25 integrated care system strategies, Chambers et al. [12] found that few mentioned the need to address the aforesaid inequities to improve access to end‐of‐life care.

The dominance of the Western paradigm compounds these disparities. The ‘bio‐reductionism of Western medicine… reduces the determinants of disease to physical factors in the body and neglects consideration of social influences’ [13]—this marginalises people whose first language is not English and people whose heritage is oral, rather than written tradition. Western enterprises, such as advanced care planning, underpinned by Western‐based ethics and values of individual autonomy and self‐determination, often exclude groups that hold different values, positioning them as ‘barriers to be overcome’ [14].

Addressing the known inequities in the quest towards integrated palliative care remains elusive. Thus, the aim of this article is to report how an innovative methodology—POSH‐VRE, which combines positive organisational scholarship in healthcare with video‐reflexive ethnography—was used to learn about brilliant palliative care from and with Syrian, Bhutanese, and African communities. The article describes how engagement with community leaders and key informants of different cultural groups was bolstered to promote ‘people‐centred integration’ [15]. However, before explaining how the study was conducted and the lessons learned, a description of brilliant palliative care is warranted, given that it might not be as familiar as other approaches, such as patient‐centred care and integrative palliative care.

To redress the scholarly preoccupation with gaps [16], issues [17], and problems [18] in palliative care, brilliant palliative care represents a purposeful reorientation to that which exceeds expectation [19]. Palliative care discourse primarily focuses on all that is wrong with it. Accounts of trials and tribulations can be readily sourced from academics [20, 21], international bodies [22], and journalists [23]. While informative, such discourse silences the experiences that exceeded expectations—experiences that mattered. Conceptually, brilliant palliative care differs from alternative approaches. For instance, while patient‐centred care and integrative palliative care largely aim to enhance a patient's ‘functional life’ [24] and their ‘quality of life and well‐being’ [25], respectively, brilliant palliative care is not tied to these intentions—instead, it is a relational experience that exceeds the expectation of those who experience or witness it [26]. Brilliant care can be unconventional and serendipitous and does not necessarily represent business as usual within a service or a sector. Furthermore, brilliant care is uplifting, inspiring and/or energising [27]. For instance, Collier et al. [28] found that brilliant palliative care is exemplified by ‘anticipatory aptitude and action; a weave of commitment; flexible adaptability; and/or team capacity‐building’. Similarly, Dadich et al. [29] concluded that brilliant community‐based palliative care ‘largely involved maintaining normality in patients’ and carers' lives’.

Building on previous research to establish the constituents of brilliant palliative care [30], this study aimed to engage with culturally and linguistically diverse communities to understand and promote brilliant palliative care with these communities. The previous research involved the methodology of POSH‐VRE. Although details on this methodology can be sourced elsewhere [27], the academic and clinician researchers captured video recordings of palliative care, as it was delivered to patients and their family members in hospitals that provided acute care, and then analysed the recordings during reflexive sessions to identify and examine moments of brilliant care. Cognisant that this research did not explicitly reflect the perspectives of culturally and linguistically diverse communities, the academic and clinician researchers engaged with culturally and linguistically diverse communities to harness their expertise. Their approach was inspired by Merten's transformative research [31] and the VRE guiding principle of research as care [32]. Specifically, they regularly engaged in critical reflection and reflexion [33] to attune to and address differences in status, power, and control and their effects on relationships and practices.

## 2 Materials and Methods

### 2.1 Participants

The researchers (A.D., A.C., G.C., C.J., and P.L.) built on an established relationship between the community leader of the Bhutanese Australian Association of South Australia (K.D.) and the then‐executive officer of a local health network volunteer organisation (P.L.). K.D. introduced the researchers to two additional community leaders of new and emerging culturally and linguistically diverse communities in Adelaide, Australia (I.Z. and D.A.). In turn, these community leaders invited the involvement of key informants from their communities—namely, the Bhutanese, African, and Syrian communities. The selection of the key informants followed due consideration of gender, age, experience, and—for the African groups—cultural diversity. As new and emerging communities, they have ‘recently arrived in Australia and are increasing in number. They… [represented] humanitarian entrants, asylum seekers, skilled migrants or part of the family stream of entrants’ [34].

### 2.2 Setting

This study was conducted across three communities in Adelaide, Australia, that represented Bhutanese, African, and Syrian communities. This was supported by the following not‐for‐profit organisations: the Bhutanese Australian Association of South Australia, the African Women's Federation, and the African Men's Foundation in Northern Adelaide.

### 2.3 Data Collection

Following the researchers' description of the study and its rationale, not all the community leaders were immediately receptive to their invitation. Some community leaders had grave reservations about a study on palliative care. Unfamiliar with the concept, they largely associated palliative care with services that accelerated death.

Over several conversations, the researchers and community leaders (K.D., I.Z., and D.A.) developed a better understanding of each other's concerns and interests. The researchers came to appreciate how palliative care services were largely absent from the Bhutanese, African, and Syrian communities, while the community leaders recognised that palliative care seeks to ‘improve… the quality of life of patients and that of their families who are facing challenges associated with life‐threatening illness, whether physical, psychological, social or spiritual’ [1]. With these changed and dynamic understandings, the researchers and community leaders considered how to engage with culturally and linguistically diverse communities to promote brilliant palliative care.

To learn from and with the three communities, the community leaders kindly agreed to invite community members to contribute to this study. Rather than use conventional recruitment methods, such as flyers or emails, they purposively invited key informants and/or other community leaders from their own communities to engage in small group discussions.

Through the course of community engagement, 14 key informants agreed to contribute to the study. They included individuals from the Bhutanese (women = 1; men = 4), African (women = 2; men = 4 from South Sudan, the Democratic Republic of the Congo, Burundi, Sierra Leone, Ivory Coast, and Somalia) and Syrian (women = 1; men = 2) communities.

Key informants were invited to participate in one of three focus groups, with one focus group for each community (*n* = 13) or an interview (*n* = 1) to accommodate their availability. Facilitated by a researcher and/or a community leader, the focus groups and interview transpired for approximately 60 min, providing an opportunity to critically consider: understandings of palliative care; conceptualisations of a good death [35]; how palliative care was typically enacted in their communities; who was (not) typically involved; how they were typically involved; why; the associated effects for individuals, their family members, and their communities; as well as the relationship between culturally and linguistically diverse communities and public palliative care services, akin to that offered by the acute care hospital. To aid discussion, participants were invited to review video recordings captured during the previous study. The video recordings prompted discussion, offering a respectful way to discuss the potentially sensitive topic of palliative care, and ‘promote critical dialogue and knowledge about important issues’ [36]. The focus groups and interview were captured via a video recording for analysis.

### 2.4 Ethical Considerations

Following clearance from the relevant ethics committees (reference number: HREC/18/CALHN/750), the community leaders and key informants of the Bhutanese, African, and Syrian communities were invited to contribute to this study. The conduct of this study was informed by national guidelines [37] and situated ethics [38]. Thus, although informed, written consent was obtained from all participants, participants were regularly invited to consider: whether they wished to continue their involvement; how the discussion should proceed; and the associated implications of engaging.

### 2.5 Data Analysis

Having viewed footage from the previous study, community leaders requested that the video‐recorded focus groups and interview be professionally produced as audio–video resources to disseminate these within their communities, facilitate discussion within their communities about palliative care, and educate and inform local service providers about cultural preferences. The audio–video resources were produced iteratively. Led by AD, the researchers collaboratively analysed the footage via six recursive phases of: familiarisation; coding; initial theme generation; theme review and development; theme refinement, definition, and identification; as well as reporting [39]. Timestamps that epitomised the themes were provided to video producers, who created a montage of video clips, which were produced into audio–video resources. This process was guided by the following interrelated lines of inquiry to establish how engagement with community leaders of different cultural groups was bolstered to promote ‘people‐centred integration’ [15]—namely, how was palliative care understood and perceived; and how could the care of people of culturally and linguistically diverse communities who experience a life‐limiting illness be improved? Further edits were made based on continuous feedback from the community leaders who reviewed the audio–video resources.

## 3 Results

### 3.1 Understandings and Perceptions of Palliative Care

Despite the cultural differences between the Bhutanese, African, and Syrian communities, the participants from these communities held similar views about specialist palliative care services, whereby it was largely deemed to be ‘a foreign idea’ (*African participant*). Collectively, they advised that there was no comparable system of health services in their birth country that provided care to people with a life‐limiting illness—this was chiefly the realm of family members and the community:

In Bhutan, our elderlies, whether they're sick or aged… they're taken to the hospital. In the last days… you take them home.

(Bhutanese participant)

It is a difference between Syria to Australia. In Syria, there is no services like this.

(Syrian participant)

For some participants, death was a taboo topic, representing ‘a no‐go area’ (*African participant*). For instance, relative to the Bhutanese and Syrian participants, African participants shared how views of death and dying in their communities were informed by traditional systems:

with the African traditional system, in Zimbabwe, when someone dies, the following day, people will go to a witchdoctor who then identify who caused the death. So even if someone is 150, 120 years old, may be a medical complication, the following morning, it's called ‘gaté’, so they go and then that witchdoctor will blame someone, most probably in the family… so death, in the African system… someone has to be blamed… there may be a ritual to find our who caused the death. Initially, there is a bit of politics within the family.

(African participant)

For some of these African participants, it was deemed disrespectful to broach the topic. This was partly because it was associated with the belief that verbal discussion might precipitate death:

people don't think that they need to say to somebody when he's passing because there is a belief that this life has been provided by God and it's the God who has taken the life whenever he want. So… telling the person… ‘You're going to die this day’… if you are part of the same culture, you will be in trouble. They will say, ‘Maybe you want to poison him. Maybe you are the one preparing his death’ because you cannot say when somebody's dying… even though… the doctor will know, they will just tell to people around that person, but not to the person itself because the person itself, they don't think it's appropriate for somebody to say and sometimes… by saying that, that can shorten his life because of the stress.

(African participant)

Perhaps partly due to the cultural restrictions around talking about death and dying, participant views about how palliative care was and should be enacted highlighted what was sometimes a cultural clash. Some intimated that palliative care clinicians informed patients when they would die—a practice that some deemed to be inappropriate. While doctors were held in high regard, they were said to contravene cultural or religious protocol by informing the patient of their prognosis. This information, they said, was to be communicated to family members, rather than to the patient. In contrast, communication with the patient was to remain hopeful and encouraging, lest their spirit be dampened:

We should not say to everybody that he is going to die before knowing that what he feel or what she feel… maybe he's upset or maybe have some kind of psychological effect… after that news, he may feel disaster.

(Bhutanese participant)

In Syria, if someone is sick, you will not go to tell him you'll die, even if you know he's got terminal; you will not tell him, even as a doctor… you might tell the family, but you will not actually go and say to him, ‘Well, I'm sorry… you probably only last for a few month’… Even though that the family might know and you might know and he might know, but it's considered against culture and against respect to say. And in fact, what you will say to him is, ‘Well, you're quite unwell; we hope from God that you'll get better’… whereas, in Australia, we do tell people.

(Syrian participant)

Unlike the Bhutanese and African participants, Syrian community members highlighted the need to avoid assumptions about particular individuals, communities, or cultures. For instance, for some of the most recent Syrian immigrants to South Australia, experiences of war made death, dying, and loss a harsh reality. These experiences challenged simplified notions that talking about death was ‘taboo’. Yet another Syrian participant of a different migrant generation explained how matters of death and dying were not necessarily discussed openly:

We thinking about the death everyday… Especially in the war… Everyone lost some of his family; everyone.

(Syrian participant)

In here… they don't talk about death. I remember when I was young… if something mentioned… someone died, [my mother]… would just change the topic and if you want to talk about something like a will… that's completely off… you do not dare mention it… you don't talk about it. It's unspoken… Some of the cultural things is that, if you talk about it, as if you're bringing a bad… thoughts, or bad expectation for the future.

(Syrian participant)

This is not to suggest that the Syrian participants had no understanding of palliative care services. They understood that it was offered to people whose illness was incurable and were expected to die. Participants, however, often conflated palliative care with end‐of‐life care. This typically involved demonstrations of assurance and empathy and surrounding the patient with the familiar—this included the home environment and family members. Such comfort was often associated with a good death:

If someone has a terminal illness, we'll say, ‘There are services available there to help you and the family to go through this terminal illness and one of them will be the palliative care’, which basically translate in Arabic words as in the care before death.

(Syrian participant)

A good death with your family, if your family around… you… at home.

(Syrian participant)

Focus group discussions provided a forum whereby common misconceptions of palliative care and palliative care clinicians were voiced. For instance, palliative care was sometimes seen as the antithesis of its central aim to help people live well with dignity:

A palliative is seen as administering a medication that gradually diminish the person and the person will die slowly.

(African participant)

Some participants spoke of the tension between the cultural expectations they were accustomed to and those of the Western paradigm. For instance, the participants collectively reinforced the importance of family members in palliative care—it was their duty to care for ill family members and was engrained into their collective identity. However, some participants intimated that, at times, clinicians falsely assumed that family involvement relegated their clinician role. As such, participants reinforced the need to avoid assumptions about the needs, preferences, and customs of patients and their family members:

In Bhutan, there is no system like that because… the… family will look after the sick people… we are farmers… everyone has to go to the farm to work whole day and at the night time when they will get time, they come and sit 24 h and whole day and night… one of the family members of the home has to give care… he will come and read some of the holy scriptures, give some of the assurance… that sympathy and empathy will give help to that person.

(Bhutanese participant)

The main issue for the Syrian community… is the explanation and the accessibility… I haven't come across anyone saying ‘Well, I don't want to get those services’… who doesn't want someone to help him?… of course, that will not replace the role of the family – that's why we say… in addition to what's available to families and friends and religious figures… you need to make sure your message is clear and it's well understood. People might nod their head, not because they understand what you tell them; it's because they're feeling shamed if they say, ‘We didn't understand’ because they look stupid, so they say, ‘Yep, yep, yep’. ‘You understand?’, ‘Yep, yep, yep’ and then they go out – ‘What should we do now?’ and I come across this quite often… We need to improve on the communication… If family want to help… They still need your support; they still appreciate your presence, and they still like to interact with you. The fact that family is there, does not mean that you basically step back… it means that you try to learn a little bit about different cultures.

(Syrian participant)

With strong familial connections, participants noted the reliance on verbal communication—this extended to the needs and preferences of people with life‐limiting illnesses. For instance, the cultural groups represented in this study did not typically rely on advanced care directives or wills—sometimes their mere mention was taboo. Instead, the person with a life‐limiting illness would verbally communicate their preferences to an appropriate family member, like their eldest child:

In our culture, we don't write things down, parents, grandparents; we don't read, write things down. So, the eldest pass information, whether it's about heritage, we don't have wills to write. If it is property that is hidden somewhere, there's no record; it's only kept in the mind. So, the parents must, at the point of death, that is where you get most information and also the other advice.

(African participant)

Given the centrality of the family and community in the care of people with life‐limiting illness, the participants identified several opportunities to integrate Western approaches to palliative care with cultural preferences. These, in turn, would promote brilliant palliative care—the type of care that exceeded their expectation.

### 3.2 Improvement Opportunities

Collectively, the participants highlighted three key opportunities to improve the care of people from culturally and linguistically diverse communities who experience a life‐limiting illness. These included: the avoidance of generalisations; prioritising the needs and preferences of cultural groups; and leveraging the community network. Each is addressed in turn.

Some participants recognised how generalising culturally and linguistically diverse communities was antithetical to palliative care and public health, more broadly. Assuming that the needs and preferences of different cultural groups were similar or worse still, synonymous, failed to appreciate their differences. Even reference to the label, ‘culturally and linguistically diverse’, was isolating—it implied uniformity among different cultures and that this single group needed to be ‘othered’. Such gross generalisations demoted the importance of a patient‐centred approach to palliative care:

you are put in a box… they call them the CALD [culturally and linguistically diverse] community… it shouldn't work like that… Yes, they lean toward one area more than you or less than you, but that doesn't mean that the general rule don't apply to them… Look at the word, CALD… When I first learnt about it… I thought it was COLD… are these communities like, a bit cold, not warm?… Boxing people in different… boxes… it can help sometimes for certain… services, but it will not be a way to go to manage things… Culture should not be barrier. The barrier is that you are not trying to understand, not the culture itself… we're humans… Please stay away from boxing people… because that [has]… far‐reaching consequences on managing so many conditions… they will affect the way the services are provided, the way the response will be.

(Syrian participant)

There's this element of generalisation that Africa seemed to be portrayed as a country in itself… It's a continent with over fifty countries with over 300… cultures and different belief systems.

(African participant)

Participants also emphasised the importance of respecting the preferences of members of cultural groups. Rather than expecting them to approach palliative care services, it was important for clinicians to proactively engage with different cultural groups and the family members of people with a life‐limiting illness. This might involve: outreach efforts to organisations that represent different cultural groups to foster relationships; ensuring palliative care services include culturally diverse staff members to optimise relatability; and demonstrating deference to family members to address power imbalances, seek their advice, and work with the family. Relative to the Bhutanese and Syrian participants, African participants indicated that these strategies would serve to promote ‘cultural intelligence’ (*African participant*):

go where there is African community organisation, like Independence Day for the Somali people… and bridge this idea so that people become aware that it exists… because with the level of deprivation in Africa, the family will take over, the family will support and that's what people [need to] know… when they are here.

(African participant)

If you consult the family, they will let you know who's in charge… listen to the patient who's the key person.

(African participant)

Given the strong connections within cultural groups, one participant suggested leveraging the community network. Akin to palliative care ambassadors [40], she noted the effectiveness and efficiency of training and supporting members of the communities to address the queries and concerns of fellow community members:

What you can do to make it easier, have key people from different communities, educate them and then once they understand, they spread the word within the community. So, that could work better than come and talk when you don't have someone key in the community who accept, who understand, who explain to the community.

(African participant)

## 4 Discussion

As per the study aim, this study demonstrates how reciprocal understandings of palliative care were facilitated using POSH‐VRE. POSH‐VRE served as a potentiation methodology [41] to build connections between palliative care services and prospective service recipients. As the partnership grew between the researchers and cultural communities, opportunities arose to probe matters of culture, palliative care, death, dying, and loss, which were hitherto unexplored, collaboratively. By valuing authentic engagement, the study helped to build foundations towards integrated palliative care [3].

Reflecting previous research [42, 43], this study suggests that members of the Bhutanese, African and Syrian communities who contributed to this study valued culturally appropriate palliative care—this included the active involvement of family members. Conversely, they noted the need to avoid assumptions about how cultural groups wish to be referred to and offered care. This finding aligns with Adusei‐Asante and Adibi's [44] critique of the term, culturally and linguistically diverse, noting that it ‘others, racially profiles, stereotypes, homogenises and inferiorises minority groups to whom the label is applied’. Through ongoing engagement, the researchers and members of the cultural communities learnt how normative assumptions and practices —including an open awareness of dying [45], which is a central tenet of palliative care, and the associated expectations of writing down end‐of‐life decisions and wishes— might contravene integrated palliative care [3].

This study also revealed participants' commonly held, although not unique misconceptions about palliative care and the associated services. These included the conflation of palliative care with the hastening of death. In turn, such beliefs can obstruct timely access to palliative care services [46].

To promote palliative care that exceeds expectations for people of culturally and linguistically diverse communities, the participants identified three opportunities—namely, avoiding generalisations, prioritising the needs and preferences of cultural groups, and leveraging the community network. Inspired by these strategies, the researchers and community leaders pursued a suite of activities to promote palliative care by fostering integration between culturally and linguistically diverse communities and palliative care clinicians. These included: producing audio–video resources for palliative care clinicians on culturally intelligent palliative care; producing audio–video resources for members of the three cultural groups represented in this study to demystify palliative care; hosting a panel discussion with palliative care clinicians, organised by and for members of African communities, to foster mutual understanding about palliative care; hosting a public forum; and delivering a presentation at a public tertiary health service. The public forum and the presentation both involved palliative care clinicians and members of the three cultural groups to demonstrate how to enact integrated palliative care.

Integration is largely conceptualised as the assimilation of specialist palliative care services and other medical specialities, much to the neglect of family or community involvement. In their scoping review of integrated palliative care, Mondejar‐Pont et al. [47] concluded that integrated palliative care provides patients with an early palliative care intervention. In other words, integration tends to be biomedically framed, often conceived in organisational and service provider terms. Yet—as Hughes et al. [4] found—this study demonstrated that integration is relational and contextual, rather than a fixed or linear intervention. Mondejar‐Pont et al. [47] also found that integrated palliative care is intended to be ‘centred on patient needs’.

Despite the importance of the findings presented in this article, it is important to note three methodological limitations. First, given the recruitment approach, which involved building on an established relationship between the community leader of the Bhutanese Australian Association of South Australia and the then‐executive officer of a local health network volunteer organisation, there are no claims that the findings represent the views of all people from Bhutanese, African, and Syrian communities. Second, the cross‐sectional study design and reliance on self‐reported data make the findings specific to the historical context in which the study was undertaken, limiting transferability. Third, given the use of reflexive thematic analysis [39], alternative analytical approaches—like a lexical analysis, which involves the use of software to establish how words travel together [48]—might have culminated with different findings.

While the aforesaid methodological limitations are noteworthy, so too are the two key strengths of this study. First, given that culturally and linguistically diverse communities are poorly represented in palliative care services and related research [11, 43], the focus of this study and the associated findings represent an important step to addressing inequities within the health system. Second, this article illustrates a fruitful process to engage with culturally and linguistically diverse communities to promote palliative care that exceeds expectations.

## 5 Conclusion

The findings presented in this article have implications for research, practice, and policy regarding palliative care. Integrated palliative care requires broad public engagement [49], not solely the engagement of (prospective) recipients of palliative care or those who deliver or manage palliative care services. Here, palliative care researchers, service providers, service managers, and policymakers might shift their attention to a whole‐system approach, where ‘Integrated care… embraces public health to support both a population‐based and person‐centred approach to care’ [15]. This is noteworthy given the insights shared by members of the Syrian, Bhutanese, and African communities—specifically, they noted the dilemma that some aspects of palliative care can be construed as inappropriate for particular reasons. For instance, its very acknowledgement might be perceived as diminishing the patient; different cultures have different understandings of a good death; clinicians might demonstrate timidity, inappropriately retreating when there is family involvement; and in some situations, written information might be inadmissible— although beyond the scope of this study, this finding in particular warrants further examination, given the legal systems in many Western nations, which rely on written artefacts. A whole‐system approach might help to engage with and negotiate these dilemmas. This kind of integration reflects the World Health Organization's public health strategy for palliative care, written back in 2007 [50]—this strategy included: education for the public as well as service providers; palliative care services at all levels; appropriate policies; and the availability of essential medicines. Integration characterised in this way aligns with ‘new’ public health approaches to palliative care—that is, an approach that focuses on wellbeing as well as health, health promotion, community partnerships, and civic involvement [51]. Accordingly, matters of integration should (partly) be determined by the patients, family members, and communities for whom integration seeks to serve. That is, for integrated palliative care to be realised, a shift in focus is required to partner with communities on addressing issues and matters of importance to them.

## Author Contributions

**Ann Dadich:** conceptualisation, methodology, writing–review and editing, writing–original draft, formal analysis, investigation, data curation. **Gregory Crawford:** writing–review and editing, funding acquisition, project administration. **Peter Laintoll:** writing–review and editing. **Issac Zangre:** writing–review and editing. **Kamal Dahal:** writing–review and editing. **Dalia Albrezi:** writing–review and editing. **Cathie Jeffs:** writing–review and editing, data curation. **Aileen Collier:** conceptualisation, methodology, writing–review and editing, writing–original draft, formal analysis, investigation, data curation, project administration.

## Ethics Statement

This study was approved by the Central Adelaide Local Health Network Human Research Ethics Committee (reference number: HREC/18/CALHN/750).

## Consent

All participants indicated informed consent.

## Conflicts of Interest

The authors declare no conflicts of interest.

## Data Availability

The authors have nothing to report.
